# Editorial: Chloride homeostasis in animal cell physiology

**DOI:** 10.3389/fphys.2023.1227565

**Published:** 2023-06-05

**Authors:** Jinwei Zhang, Anna-Maria Hartmann, Jiangtao Guo

**Affiliations:** ^1^ Institute of Cardiovascular Diseases, Xiamen Cardiovascular Hospital Xiamen University, School of Medicine, Xiamen University, Xiamen, Fujian, China; ^2^ State Key Laboratory of Chemical Biology, Center for Excellence in Molecular Synthesis, Shanghai Institute of Organic Chemistry, Chinese Academy of Sciences, Shanghai, China; ^3^ Institute of Biomedical and Clinical Sciences, Medical School, Faculty of Health and Life Sciences, University of Exeter, Hatherly Laboratories, Streatham Campus, Exeter, United Kingdom; ^4^ Division of Neurogenetics, Faculty VI, School of Medicine and Health Sciences, Carl von Ossietzky University Oldenburg, Oldenburg, Germany; ^5^ Research Center for Neurosensory Sciences, Carl von Ossietzky University Oldenburg, Oldenburg, Germany; ^6^ Department of Biophysics, Zhejiang University School of Medicine, Hangzhou, China; ^7^ Department of Neurology of the Fourth Affiliated Hospital, Zhejiang University School of Medicine, Hangzhou, China

**Keywords:** chloride ion channels and cotransporters, cellular Cl-volume regulation, Cl-homeostasis, creatine balance, exocytosis, mucus secretion, drug development

Chloride (Cl^−^) homeostasis is a critical aspect of animal cell physiology that is often overlooked, but it plays a vital role in maintaining the balance of charge within cells. Chloride ions, along with sodium (Na^+^) and potassium (K^+^), are responsible for osmotic pressure and acid-base balance and determine fundamental biological functions in all tissues. Cl^−^ is not in electrochemical equilibrium in most cell types, and its regulation involves the coordination of several processes. Cotransporters and exchangers utilize electric potential and/or chemical gradients to move two or more protons and ions in the same or opposite directions across the cell membrane. Impaired Cl^−^ transport affects diverse processes ranging from neuron excitability to water secretion, which underlie pathological conditions such as epilepsy, deafness, imbalance, brain edema and ischemia, pain and neurogenic inflammation, hypertrophy, or heart failure-induced remodelling, chronic kidney disease and cystic fibrosis, *etc.* Therefore, further investigation to explore the molecular mechanisms of nociception occurring via membrane-bound Cl^−^ ion channels or co-transporters is vital for understanding cellular physiology and pathophysiology, as well as for developing a new class of therapeutics.

**FIGURE 1:**
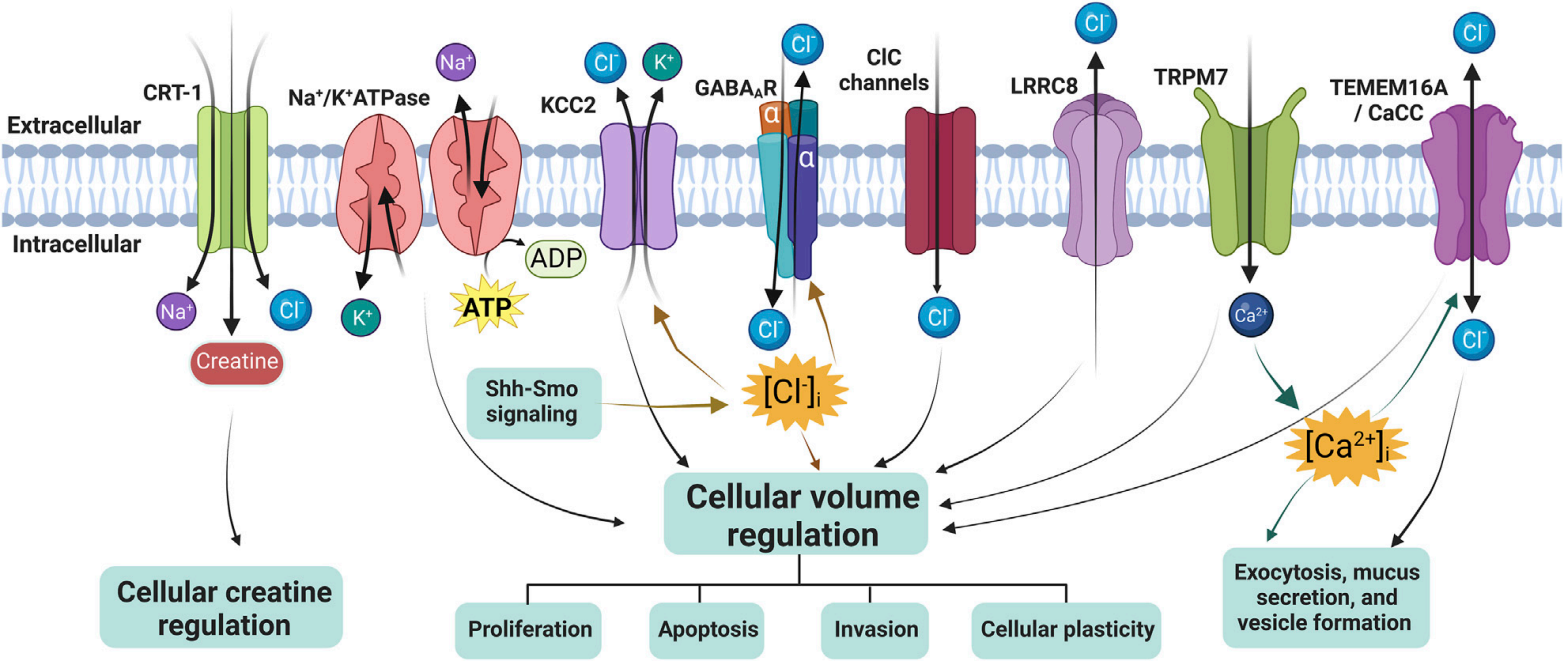
Overview of chloride ion channels, ion cotransporters, and creatine transporter in regulating cellular volume or creatine. CRT-1, creatine transporter 1; KCC2, K^+^-Cl^−^ co-transporter 2; ClC channels, Cl^−^ channels; LRRC8, leucine-rich repeat-containing 8; TMEM16A, transmembrane protein 16A; TRPM7, transient receptor potential cation channel subfamily M member 7; CaCC, Ca^2+^-activated Cl^−^ channel, and GABA_A_R, γ-aminobutyric acid (GABA) _A_ receptor. The diagram was created using BioRender.com.

The Research Topic includes two original research papers and three reviews from prominent researchers in the field and provides readers of the journal with recent results or summaries in the area of mechanisms of chloride ion channels and co-transporters in cellular Cl^−^ volume regulation, Cl^−^ homeostasis, creatine balance, exocytosis, mucus secretion, and formation of extracellular vesicles (Figure 1), as well as new strategies for drug development.

The volume-regulated anion channel (VRAC) is present in various cell types and plays a crucial role in facilitating swelling-induced Cl^−^ currents (*I*_Cl_, swell) for cell volume regulation. To date, VRAC primarily consists of three main components: 1) a family of leucine-rich repeat containing 8 (LRRC8) proteins, which includes five members (LRRC8A-E); 2) the solute carrier organic anion transporter family member 2a1 gene (*SLCO2A1*), responsible for encoding organic anion transporting polypeptide (OATP) 2A1, also known as prostaglandin (PG) transporter, is a high-affinity prostanoid carrier; and 3) transmembrane protein 206 (TMEM206), an essential subunit of the acid-sensitive outwardly rectifying (ASOR) anion channel. Most of these channels have been identified as crucial core or pore-forming elements in various anion channels. Dysregulation of them can lead to cellular swelling or shrinkage, which can have significant consequences for cell function and survival. In their comprehensive review, Okada et al. discuss the current understanding of these channels with respect to their molecular identities, functional properties, structural features, and physiological roles. The review summarizes that these anion channels play a crucial role not only in essential physiological cell functions but also in pathological conditions by regulating/dysregulating cell volume and releasing organic signals.

KCC2, a K^+^-Cl^−^ co-transporter mainly found in neurons, is a vital molecule that regulates Cl^−^ extrusion and establishes the resting level of (Cl^−^)_i_ in both developing and mature mammalian neurons, thereby tuning the strength and polarity of GABA_A_ receptor-mediated transmission (Tillman and Zhang, 2019). One of the factors influencing the functioning of KCC2 and the development of inhibitory circuits is Smoothened (Smo), which acts as a transducer in the receptor complex of the developmental protein Sonic Hedgehog (Shh) (Delmotte et al., 2020). In this timely review, Hamze et al. delve into the latest research on how the Shh-Smo signaling pathways affect Cl^−^ homeostasis by regulating KCC2 membrane trafficking, which, in turn, impacts inhibitory neurotransmission and network activity during postnatal development. Indeed, this new discovery opens up new avenues of research on Shh and GABA for physiological research, as well as for identifying potential pharmacological targets against neuropsychiatric and neurological diseases.

Maintaining appropriate levels of Cl^−^ through Cl^−^ channels is crucial for the normal functioning of the central nervous system (CNS). These channels consist of voltage-gated Cl^−^ channels (ClC family), such as ClC-1 and ClC-2; Ca^2+^-activated Cl^−^ channels (CaCCs), including Anoctamins 1 and 2 and Bestrophin1 (Best1); Cystic fibrosis transmembrane conductance regulator (CFTR); VRAC; GABA_A_-gated Cl^−^ channels (GABA_A_ receptor); and maxi anion channels (MAC). Wang and Choi have reviewed five families of Cl^−^ channels expressed by neurons and glia and their role in the pathogenesis of CNS disorders. They have also summarized the small molecule Cl^−^ channel modulators currently used as potential therapeutics in CNS disorders. Additionally, a possible strategy for optimizing drug permeation through the blood-brain barrier (BBB) has been suggested.

The creatine transporter (CRT) is a Na^+^- and Cl^−^ -dependent transporter that is primarily responsible for transporting creatine across the cell membrane. CRT-1, a member of the *SLC*6 family (*SLC*6A8), is believed to play a crucial role in supplying creatine to skeletal and cardiac muscle, as well as the nervous system, where it acts as a precursor to phosphocreatine, a substance that replenishes spent ATP. Mutations in CRT-1 are implicated in several neurological disorders (Bizzi et al., 2002; Hahn et al., 2002), underscoring the importance of gaining insight into its mechanism and the effects of mutations on its function. However, our understanding of its molecular mechanisms lags far behind that of other well-studied *SLC* transporters and remains a critical question to be answered. Farr et al. used whole-cell patch clamp electrophysiological recordings on CRT-1-expressing HEK293 cells in combination with mathematical modelling to develop a kinetic model of CRT-1-mediated transport. They concluded that to maintain cytosolic creatine concentrations as observed, CRT-1 must destabilize binary complexes rather than ternary ones. Additionally, they provide a plausible explanation as to why neurons, heart, and skeletal muscle cells require a transporter that releases creatine, facilitating the quick balancing of creatine levels within these cells.

The Ca^2+^-activated Cl^−^ channel TMEM16A and the Cl^−^ permeable phospholipid scramblase TMEM16F are examples of Cl^−^ channels that could have an impact on the intracellular Cl^−^ concentration ([Cl^−^]_i_), potentially serving as an intracellular signal. If TMEM16A expression is lost in the airway, it can lead to a significant increase in the population of secretory cells such as goblet and club cells, resulting in the differentiation of the airway epithelium into a secretory one (He et al., 2020). Centeio et al. evaluated the role of TMEM16A and TMEM16F, as well as the Notch pathway, in airway epithelial cell differentiation using epithelial cultures and conditional knockout models. Their data found that TMEM16A/F are important for exocytosis, mucus secretion, and formation of extracellular vesicles (exosomes or ectosomes). However, their present data do not support a functional role of TMEM16A/F in Notch-mediated differentiation of BCi-NS1.1 cells towards a secretory epithelium.

Overall, this Research Topic summarizes important findings and recent research progress related to chloride ion channels and co-transporters, their mechanisms of regulation, and new strategies of drug development.
